# Plasma gamma-glutamylglycine predicts the seroconversion of hepatitis B e antigen in patients with chronic hepatitis B

**DOI:** 10.3389/fimmu.2026.1873056

**Published:** 2026-06-08

**Authors:** Ze-Hua Zhao, Zheng-Jie Qu, Xin-Hua Guo, Xue-Qi Yang, Qi An, Fang-Ming Zhou, Jing-Hui Tian, Yu-Chen Fan

**Affiliations:** 1 Department of Hepatology, Qilu Hospital of Shandong University, Hepatology Institute of Shandong University, Jinan, China; 2 School of Public Health, Shandong First Medical University and Shandong Academy of Medical Sciences, Jinan, China; 3 Department of Clinical Laboratory, The Second Affiliated Hospital of Shandong First Medical University, Taian, China

**Keywords:** antiviral therapy, chronic hepatitis B, gamma-glutamylglycine, HBeAg seroconversion, metabolomics

## Abstract

**Background:**

The seroconversion of hepatitis B e antigen (HBeAg) remains difficult to be completely obtained in the treatment of hepatitis B. This study aimed to characterize the metabolic signatures of HBeAg-positive patients and identify key metabolites associated with HBeAg seroconversion.

**Methods:**

Untargeted metabolomic analysis was conducted to investigate the plasma metabolic profiles of HBeAg-positive (n=54) and negative (n=33) patients. Subgroup analyses were performed in patients with persistent HBeAg<20IU/ml level (n=33) versus those with HBeAg-negative (n=33) and patients with immune tolerant (n=8) versus immune active phases (n=7). Targeted metabolomic analysis was used to validate specific amino acids in an external cohort (n=90). The biological effects of gamma-glutamylglycine were examined by *in vitro* experiments.

**Results:**

Compared with patients who achieved HBeAg seroconversion, HBeAg-positive patients exhibited distinct metabolic profiles, with 227 differential metabolites (133 downregulated and 94 upregulated, P<0.05). KEGG pathway enrichment analysis revealed downregulated tryptophan, pyruvate, glycine, serine, and threonine metabolism, and glycerophospholipid metabolism and upregulated arginine biosynthesis, and cysteine and methionine metabolism in HBeAg-positive patients (P<0.01). Gamma-glutamylglycine and Na-L-glutamyl-aspartic acid were significantly upregulated in HBeAg-positive patients by both untargeted and targeted metabolomic analysis (P<0.05). Furthermore, a predictive model integrating plasma gamma-glutamylglycine accurately identified HBeAg seroconversion in both developing (AUC = 0.857, 95% CI: 0.765-0.923) and external (AUC = 0.780, 95% CI: 0.680-0.861) cohorts. *In vitro* assays showed that gamma-glutamylglycine contributed to the production of HBeAg in HBV-replicating cells.

**Conclusions:**

Altered amino acid metabolism is a predominant metabolic feature of HBeAg-positive patients and plasma levels of gamma-glutamylglycine are negatively associated with HBeAg seroconversion.

## Introduction

Chronic hepatitis B virus (HBV) infection poses a substantial public health burden worldwide. An estimated 296 million people are affected by HBV and approximately 15%–40% of untreated patients progress to liver cirrhosis or hepatocellular carcinoma (HCC), contributing to nearly 820, 000 HBV-related deaths each year (1). The World Health Organization has set a target to eliminate the public health threat of viral hepatitis by 2030 (2), but numerous obstacles and challenges remain (3). The pathophysiology of HBV infection is complex, and a considerable proportion of hepatitis B e antigen (HBeAg)-positive patients are immunotolerant, showing only mildly elevated alanine aminotransferase (ALT) levels and benign liver histology. However, the immunotolerant phase may eventually progress to an active phase (4). HBeAg seroconversion in patients with chronic hepatitis B (CHB) leads to a marked decrease in the HBV DNA levels and reduces the incidence of liver cirrhosis and HCC (5, 6). Although pegylated-interferon α (PEG-IFNα) and nucleos(t)ide analogues (NAs) are available for treating CHB, achieving HBeAg seroconversion is not guaranteed. PEG-IFNα therapy has been reported to induce HBeAg seroconversion in up to 32% of patients with HBeAg-positive CHB at 6 months after treatment, and the HBeAg seroconversion rate is approximately 20% in patients with treatment-naïve HBeAg-positive CHB after one year of NA therapy (7, 8). However, the exact mechanisms underlying HBeAg seroconversion remain largely unknown.

Recent studies have demonstrated metabolic alterations in liver diseases (9, 10). Metabolic alterations have been shown to contribute to immune dysfunction and disease progression in HBV-related decompensated cirrhosis and acute-on-chronic liver failure (11–13). Metabolomics enables the measurement of metabolite changes in physiological and pathological states and can thus be used to detect metabolic changes in patients with chronic HBV infection. For instance, plasma lipid metabolites can differentiate metabolic from viral chronic liver disease (14). Moreover, metabolic biomarkers can significantly enhance the prediction of occurrence and outcomes of HBV-related diseases (9). We recently identified a gut microbial metabolite that regulates CD4+ T cell immunity and promotes HBV clearance (15). These findings suggest that metabolic regulation plays a pivotal role in anti-HBV immunity and metabolic signatures have the potential to monitor clinical outcomes of CHB patients. In the present study, we aimed to characterize the metabolic signatures of HBeAg-positive patients and identify the key metabolites associated with HBeAg seroconversion.

## Patients and methods

### Study population and sample collection

A developing cohort of 87 HBeAg-positive patients, including 33 patients who had achieved HBeAg seroconversion, were recruited from March 2023 to March 2024 and an external cohort of 90 patients with HBeAg-positive CHB, including 50 patients who had achieved HBeAg seroconversion, were recruited from April 2024 to April 2025 in the Department of Hepatology, Qilu Hospital of Shandong University. All included CHB patients were diagnosed as a documented history of HBsAg positivity for more than 24 weeks, and were HBeAg positive at the time of recruitment (16). HBeAg seroconversion was defined as the loss of HBeAg and presence of anti-HBe in individuals who were previously HBeAg positive and anti-HBe negative. The immune tolerant (IT) phase was characterized by HBsAg positivity, HBeAg positivity, anti-HBe negativity, anti-HBc positivity, and normal ALT levels. The immune active (IA) or clearance phase was characterized by HBsAg positivity, HBeAg positivity, anti-HBe negativity, anti-HBc positivity, and elevated ALT levels (>40 U/L). The exclusion criteria were as follows: (1) liver diseases caused by alcohol, drugs, hepatitis C virus, hepatitis E virus, autoimmune liver diseases, or other non-HBV viruses; (2) liver cancer, extrahepatic malignancies, rheumatic diseases, or hyperthyroidism; (3) aged <18 years; and (4) being pregnant. Fasting blood samples were collected, immediately centrifuged at 1500 × *g* for 10 min, and stored at −80 °C until assay. The study protocol was in accordance with the Declaration of Helsinki and approved by the Medical Ethics Committee of Qilu Hospital, Shandong University (KYLL-202301-008-1). All participants provided written informed consent prior to enrollment.

### Demographic and clinical characteristics

We collected the following demographic and clinical characteristics: age, sex, ALT level, aspartate aminotransferase (AST) level, platelet count, white blood cell count, and HBV marker levels (HBsAg, HBeAg, and serum HBV DNA). Biochemical parameters were measured using routine automated analysers (COBAS 8000, Roche Diagnostics, Mannheim, Germany). Hepatitis B serological markers were detected using a commercially available analyser (COBAS 6000, Roche Diagnostics, Mannheim, Germany). HBV DNA was measured using a real-time polymerase chain reaction system (ABI 7300, Applied Biosystems, CA, USA), with a detection sensitivity of 100 IU/mL.

### Untargeted metabolomics analysis

Untargeted metabolomics analysis was conducted by Shanghai Luming Biological Technology Co., Ltd. (Shanghai, China). The original liquid chromatography–MS data were processed using Progenesis QI V2.3 software (Nonlinear Dynamics, Newcastle, UK) for baseline filtering, peak identification, integration, retention time correction, peak alignment, and normalisation. Compounds were identified based on precise m/z, secondary fragments, and isotopic distribution using the Human Metabolome Database, Lipidmaps (V2.3), Metlin, and self-built databases (17). The extracted data were further processed by removing peaks with missing values (ion intensity = 0) in more than 50% of samples in each group, replacing zero values with half of the minimum detected value, and screening according to compound qualitative results. A data matrix was then compiled from both the positive and negative ion data (17). Absolute quantification of candidate amino acids and peptides including gamma-glutamylglycine and Na-L-glutamyl-aspartic in plasma samples were performed using an ultra-performance liquid chromatography system coupled to a tandem mass spectrometry (UPLC-MS/MS) system. The raw data files were processed using TMBQ software.

### Targeted metabolomics analysis

Targeted metabolomics analysis was conducted by Shanghai Sensichip Infotech Co., Ltd. (Shanghai, China). MS analysis was carried out in positive ionization mode on a 5500 QTRAP mass spectrometer (AB SCIEX, Framingham, MA, USA). The chromatographic peak area and analyte retention time were extracted using Multiquant software. Based on the standards of gamma-glutamylglycine and Na-L-glutamyl-aspartic, the analyte retention time was corrected, followed by identification of the metabolites.

### Cell lines and treatment

HepG2.2.15 cells (purchased from China Center for Type Culture Collection, Wuhan, China) and HepAD 38 cells (kindly provided by Prof. Fengmin Lu, Peking University) were cultured in Dulbecco’s Modified Eagle Medium (DMEM) (Gibco, CA, USA) supplemented with 10% fetal bovine serum, 100 IU/ml penicillin, and 100 μg/mL streptomycin at 37 °C in a humidified atmosphere containing 5% CO_2_. The cells were treated with gamma-glutamylglycine (MedChemExpress, Shanghai, China) with a concentration range of 0.1-100 μM for 6 days.

### CCK-8 assay

HepG2.2.15 and HepAD38 cells were seeded in 96-well plates at a density of 5 × 10³ cells per well in 100μl medium and allowed to adhere for 24 hours. After 6-day treatment of gamma-glutamylglycine, 10 μl CCK-8 reagent (Yeasen Biotechnology, Shanghai, China) was added and the plates were incubated at 37 °C for 35 minutes. Absorbance was measured at 450 nm using a spectrophotometer, with a reference wavelength of 650 nm for background subtraction. Cell viability was calculated relative to the untreated control group.

### HBV DNA and HBeAg measurement

For HBV DNA and HBeAg measurement, cell culture supernatants were collected. HBV DNA levels were quantified using the One-Step Quantitative Detection Kit for Hepatitis B Virus Nucleic Acid (Sansure Biotech, Changsha, China) according to the manufacturer’s instructions. HBeAg levels were measured using an enzyme-linked immunosorbent assay (ELISA) kit (InTec Products Inc., Xiamen, China) following the manufacturer’s protocol. Absorbance was read at 450nm using a spectrophotometer.

### Statistical analysis

Statistical analysis was conducted using R Software (version 3.6.1, R Foundation for Statistical Computing, Vienna, Austria) and GraphPad Prism (version 7.0, GraphPad Software, Boston, MA). Quantitative data are presented as the median (interquartile range, IQR), and categorical data are presented as the number (percentage). The Shapiro–Wilk method was applied to test for normality. The chi-square test was used to compare categorical variables, whereas continuous variables were compared using the Kruskal–Wallis test for multiple comparisons and the Mann–Whitney U test for comparisons between two groups. Random forest, a machine learning method, was used to identify and prioritise biomarker candidates, which were then added stepwise into a logistic regression model to predict HBeAg seroconversion. Receiver operating characteristic (ROC) curves were generated, and the area under the curve (AUC) with 95% confidence intervals (CIs) was calculated to assess model efficacy. Two-sided P values were reported and P<0.05 was considered statistically significant.

## Results

### Characteristics of included patients

The developing cohort included 87 patients with chronic HBV infection and were divided into HBeAg-positive (n=54) and HBeAg-negative (n=33) groups based on HBeAg seroconversion. The demographic and clinical characteristics of the included patients are summarised in **Table 1**. There were no significant differences in age, sex, or biochemical parameters (ALT, AST, gamma-glutamyl transferase, alkaline phosphatase, and albumin) between the groups (P>0.05). The HBsAg level and fibrosis-4 index were also comparable between the groups (P>0.05). Similarly, HBsAg and HBV DNA levels did not differ significantly between the groups. For subgroup analysis, patients with a serum HBeAg level below 20 IU/mL were selected as the HBeAg-low group (n=11). There were no significant differences in demographic characteristics or biochemical parameters between the HBeAg-low and HBeAg-negative groups (**Table 2**). In addition, the IT (n=8) and IA (n=7) groups were subjected to metabolomic analysis and their basic characteristics are listed in **Table 3**. The external cohort included 40 HBeAg-positive and 50 HBeAg-negative CHB patients. Baseline characteristics of subjects in the external cohort are shown in **Table 1**.

**Table 1:** Baseline characteristics of HBeAg-negative and HBeAg-positive patients in developing and external cohorts.

	Developing cohort (N = 87)				External cohort (N = 90)			
Variable	HBeAg negative, N = 33	HBeAg positive, N = 54	Statistics	P value	HBeAg negative, N = 40	HBeAg positive, N = 50	Statistics	P value
Age (years)	39.00 (32.50, 48.00)	36.50 (33.00, 47.25)	811.00	0.671	38.00 (33.25, 53.75)	40.00 (34.00, 51.25)	973.50	0.829
Gender			0.02	0.886			0.63	0.427
Female	10.00 (30.30%)	16.00 (29.63%)			12.00 (30.00%)	19.00 (38.00%)		
Male	23.00 (69.70%)	38.00 (70.37%)			28.00 (70.00%)	31.00 (62.00%)		
HBsAg (IU/ml)	4574.47 (2467.35, 7629.39)	3846.57 (1846.33, 8648.52)	756.00	0.358	2442.55 (397.32, 7699.73)	3977.52 (1899.10, 7007.47)	692.00	0.012
HBeAg (IU/ml)	0.00	94.33 (25.78, 585.98)	0.00	<0.001	0.00	16.78 (10.86, 451.40)	0.00	<0.001
HBV DNA			0.64	0.424			2.60	0.107
Negative	22.00 (66.67%)	30.00 (55.56%)			28.00 (70.00%)	24.00 (48.00%)		
Positive	11.00 (33.33%)	24.00 (44.44%)			12.00 (30.00%)	26.00 (52.00%)		
ALT (U/L)	25.00 (15.00, 37.50)	27.50 (19.50, 42.25)	698.00	0.149	19.00 (15.00, 28.00)	23.00 (17.00, 34.50)	809.00	0.218
AST (U/L)	21.00 (17.50, 25.50)	24.00 (19.00, 31.75)	657.00	0.070	19.00 (17.00, 23.00)	23.00 (18.00, 30.00)	673.50	0.018
GGT (U/L)	24.00 (14.00, 29.00)	21.00 (12.00, 55.00)	842.00	0.885	19.00 (14.00, 30.00)	20.00 (14.00, 36.50)	935.50	0.866
AKP (U/L)	80.00 (65.50, 88.00)	76.00 (63.00, 87.75)	830.00	0.801	75.00 (58.00, 95.00)	82.00 (65.25, 99.75)	774.50	0.168
ALB (g/L)	48.30 (45.85, 49.40)	47.80 (44.65, 49.78)	818.5.50	0.722	47.25 (46.00, 48.60)	46.40 (45.43 48.45)	719.00	0.093
FIB-4	0.80 (0.61, 1.26)	0.76 (0.62, 1.11)	822.00	0.745	0.87 (0.64, 1.24)	0.95 (0.63, 1.24)	851.00	0.783

HBsAg, hepatitis B surface antigen; HBeAg, hepatitis B e antigen; HBV, hepatitis B virus; ALT, alanine aminotransferase; AST, aspartate aminotransferase; GGT, gamma-glutamyl transferase; AKP, alkaline phosphatase; ALB, albumin; FIB-4, fibrosis-4 index.

**Table 2:** Baseline characteristics of HBeAg-negative and HBeAg-low patients.

Variable	HBeAg<20 IU/ml, N = 11	HBeAg negative, N = 33	Statistics	P value
Age (years)	36.00 (33.00, 42.00)	39.00 (32.50, 48.00)	147.00	0.349
Gender			0.04	0.849
Female	3.00 (27.27%)	10.00 (30.30%)		
Male	8.00 (72.73%)	23.00 (69.70%)		
HBsAg (IU/ml)	1927.91 (1683.60, 3617.02)	4574.47 (2467.35, 7629.39)	87.00	0.009
HBeAg (IU/ml)	5.31 (2.26, 13.09)	0.00	0.00	<0.001
HBV DNA			4.89	0.027
Negative	11.00 (100.00%)	22.00 (66.67%)		
Positive	0.00 (0.00%)	11.00 (33.33%)		
ALT (U/L)	23.00 (16.00, 32.00)	25.00 (15.00, 37.50)	176.00	0.894
AST (U/L)	19.00 (17.00, 27.00)	21.00 (17.50, 25.50)	159.00	0.556
GGT (U/L)	14.00 (11.00, 34.00)	24.00 (14.00, 29.00)	157.50	0.521
AKP (U/L)	76.00 (66.00, 84.00)	80.00 (65.50, 88.00)	169.00	0.745
ALB (g/L)	49.30 (45.90, 50.00)	48.30 (45.85, 49.40)	143.50	0.308
FIB-4	0.70 (0.44, 1.10)	0.80 (0.61, 1.26)	154.00	0.470

HBsAg, hepatitis B surface antigen; HBeAg, hepatitis B e antigen; HBV, hepatitis B virus; ALT, alanine aminotransferase; AST, aspartate aminotransferase; GGT, gamma-glutamyl transferase; AKP, alkaline phosphatase; ALB, albumin; FIB-4, fibrosis-4 index.

**Table 3:** Baseline characteristics of immune active and immune tolerant patients.

Variable	Immune active, N = 7	Immune tolerant, N = 8	Statistics	P value
Age (years)	31.00 (28.00, 38.00)	38.00 (31.50, 48.00)	19.00	0.118
Gender			0.24	0.627
Female	2.00 (28.57%)	3.00 (37.50%)		
Male	5.00 (71.43%)	5.00 (62.50%)		
HBsAg (IU/ml)	37848.06 (3085.31, 109856.92)	43822.61 (19646.85, 73331.33)	33.00	0.845
HBeAg (IU/ml)	1433.47 (861.96, 1770.35)	1, 400.35 (723.46, 1576.97)	32.00	0.769
HBV DNA			0.00	>0.999
Positive	7.00 (100.00%)	8.00 (100.00%)		
Negative	0.00 (0.00%)	0.00 (0.00%)		
ALT (U/L)	75.00 (58.00, 210.00)	22.50 (11.00, 26.50)	0.01	0.001
AST (U/L)	54.00 (36.00, 141.00)	20.00 (18.00, 21.00)	2.00	0.001
GGT (U/L)	41.00 (31.00, 50.00)	17.50 (9.50, 22.00)	7.50	0.007
AKP (U/L)	80.00 (73.00, 96.00)	60.00 (55.75, 71.75)	8.00	0.008
ALB (g/L)	47.30 (46.20, 47.80)	47.70 (45.2, 50.25)	33.00	0.845
FIB-4	0.78 (0.44, 1.97)	0.81 (0.68, 1.02)	34.00	0.922

HBsAg, hepatitis B surface antigen; HBeAg, hepatitis B e antigen; HBV, hepatitis B virus; ALT, alanine aminotransferase; AST, aspartate aminotransferase; GGT, gamma-glutamyl transferase; AKP, alkaline phosphatase; ALB, albumin; FIB-4, fibrosis-4 index.

### Altered metabolic profiles in HBeAg-positive patients

**Figure 1:**
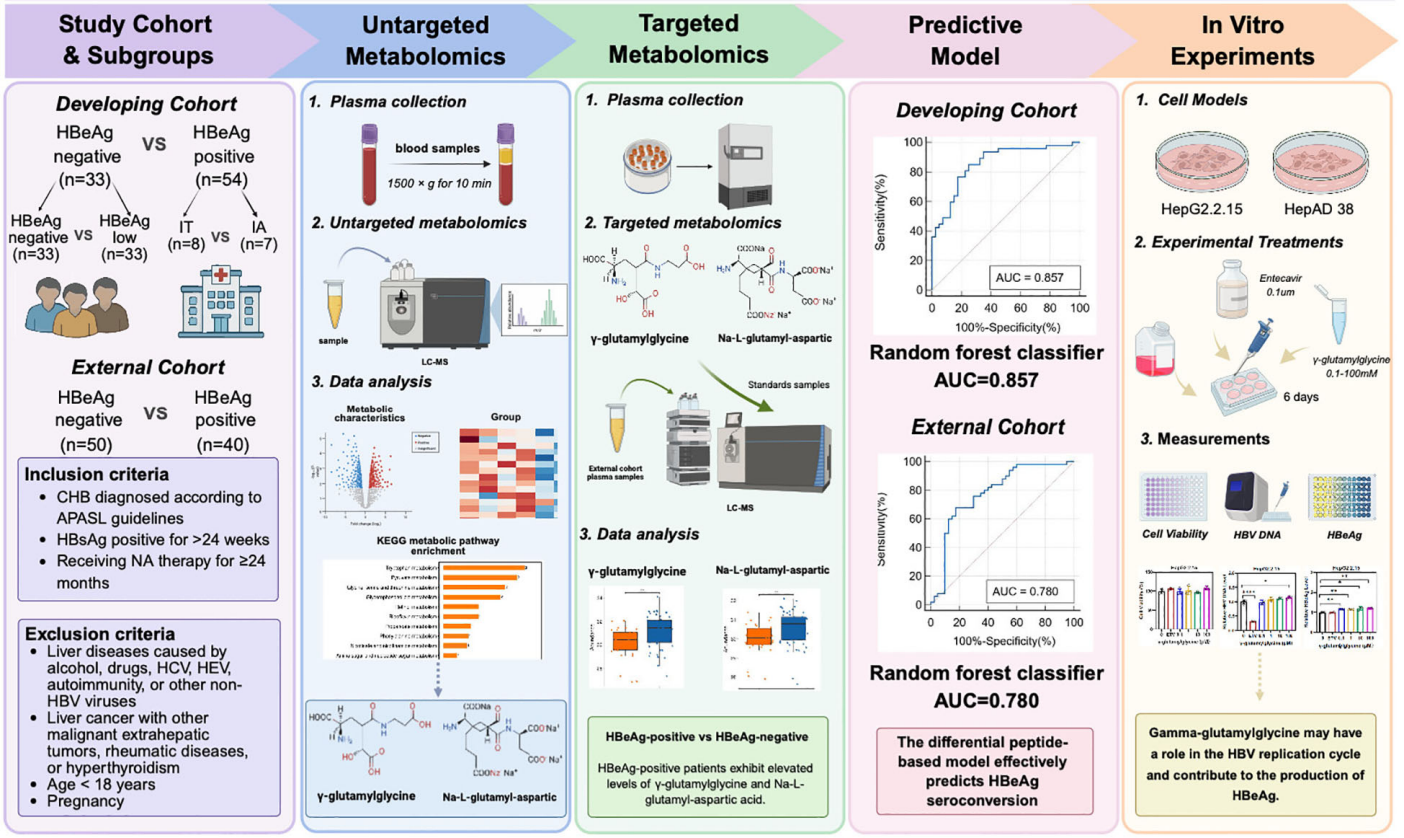
Schematic figure of the study design.

**Figure 2:**
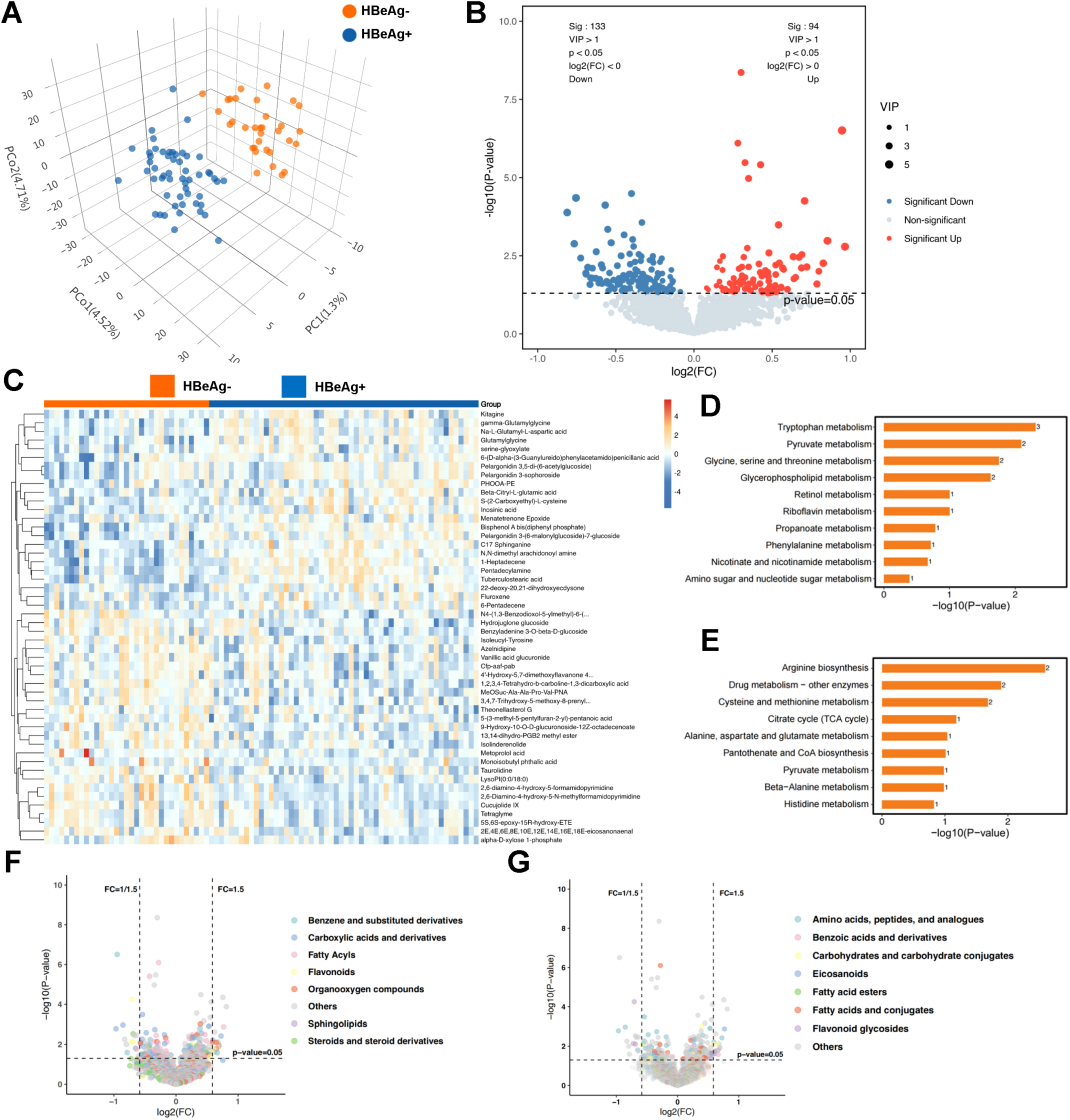
Altered metabolic pathways and metabolites in HBeAg-positive patients compared with patients achieving HBeAg seroconversion. **(A)** The OPLS-DA model of plasma metabolomic analysis for HBeAg-positive and HBeAg-negative patients. **(B)** Volcano plot showing significantly upregulated and downregulated metabolites between HBeAg-positive and HBeAg-negative groups. **(C)** Heatmap of differential metabolites between HBeAg-positive and HBeAg-negative groups. KEGG pathway enrichment analysis identified downregulated pathways **(D)** and upregulated pathways **(E)** in HBeAg-positive patients compared with HBeAg-negative patients. **(F)** Volcano plot showing significantly regulated metabolites at the class level and **(G)** subclass level.

The study design is shown in **Figure 1**. We first compared metabolic characteristics between the HBeAg-positive and HBeAg-negative groups. HBeAg-positive patients exhibited distinct metabolic profiles compared with those with those who achieved HBeAg seroconversion (**Figure 2A**). We identified 227 differential metabolites, including 133 downregulated and 94 upregulated metabolites in HBeAg-positive patients (**Figures 2B, C**). KEGG pathway enrichment analysis revealed 7 dysregulated pathways: downregulated tryptophan metabolism, pyruvate metabolism, glycine, serine, and threonine metabolism, and glycerophospholipid metabolism and upregulated arginine biosynthesis, drug metabolism, and cysteine and methionine metabolism (P<0.01, **Figures 2D, E**). Carboxylic acids, particularly amino acids and peptides, were significantly altered in HBeAg-positive patients (**Figures 2F, G**). These findings suggest that metabolic profiles in HBeAg-positive patients differ significantly from those in patients with HBeAg seroconversion, with prominent alterations in amino acid and peptide metabolism.

### Impaired amino acid metabolism in patients with a low HBeAg level

**Figure 3:**
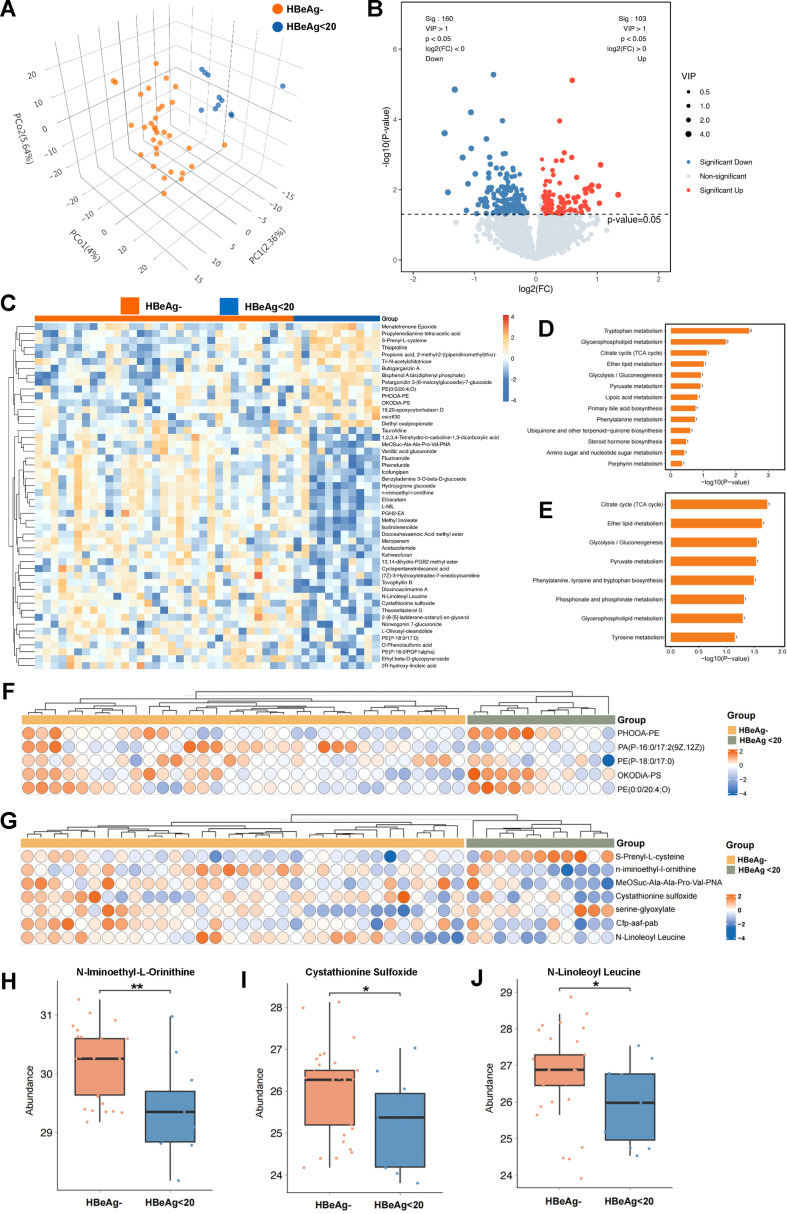
Altered metabolic pathways and metabolites in patients with low HBeAg level compared with patients achieving HBeAg seroconversion. **(A)** OPLS-DA model of plasma metabolomic analysis for HBeAg-low and HBeAg-negative patients. **(B)** Volcano plot showing significantly upregulated and downregulated metabolites between HBeAg-low and HBeAg-negative groups. **(C)** Heatmap of differential metabolites between HBeAg-low and HBeAg-negative groups. KEGG pathway enrichment analysis identified downregulated pathways **(D)** and upregulated pathways **(E)** in HBeAg-low patients compared with HBeAg-negative patients. **(F)** Heatmap showing altered glycerophospholipid species in HBeAg-low and HBeAg-negative groups. **(G)** Heatmap showing altered amino acid and peptide species in HBeAg-low and HBeAg-negative groups. **(H-J)** Plasma levels of specific amino acids and peptides were significantly altered in HBeAg-low patients. *P<0.05, **P<0.01.

Next, we investigated the metabolic characteristics of HBeAg-positive patients whose serum HBeAg level had declined to a low level (<20IU/mL) but who had not achieved HBeAg seroconversion despite long-term antiviral therapy. Compared with the HBeAg-negative group, the HBeAg-low group showed significantly different metabolic profiles (**Figure 3A**). A total of 263 dysregulated metabolites were identified, including 160 downregulated and 103 upregulated metabolites in patients with a low HBeAg level (**Figures 3B, C**). KEGG pathway enrichment analysis demonstrated that tryptophan metabolism and glycerophospholipid metabolism were significantly downregulated (P<0.01), whereas the citrate cycle was upregulated (P<0.05, **Figures 3D, E**). Given that amino acid and glycerophospholipid metabolism were also dysregulated in the HBeAg-positive group, we analyzed the significantly regulated glycerophospholipids, amino acids, and peptides in the HBeAg-low group. The results indicated that, compared with glycerophospholipids, amino acids and peptides were prominently downregulated in patients with low HBeAg levels (**Figures 3F, G**). Specifically, n-iminoethyl-l-ornithine, cystathionine sulfoxide, and n-linoleoyl leucine were the most downregulated amino acids and peptides (**Figures 3H–J**). Collectively, these results suggest that amino acid metabolism is impaired in patients with a low HBeAg level, which may contribute to the failure of HBeAg seroconversion.

### Differential metabolic characteristics of IT and IA patients

**Figure 4:**
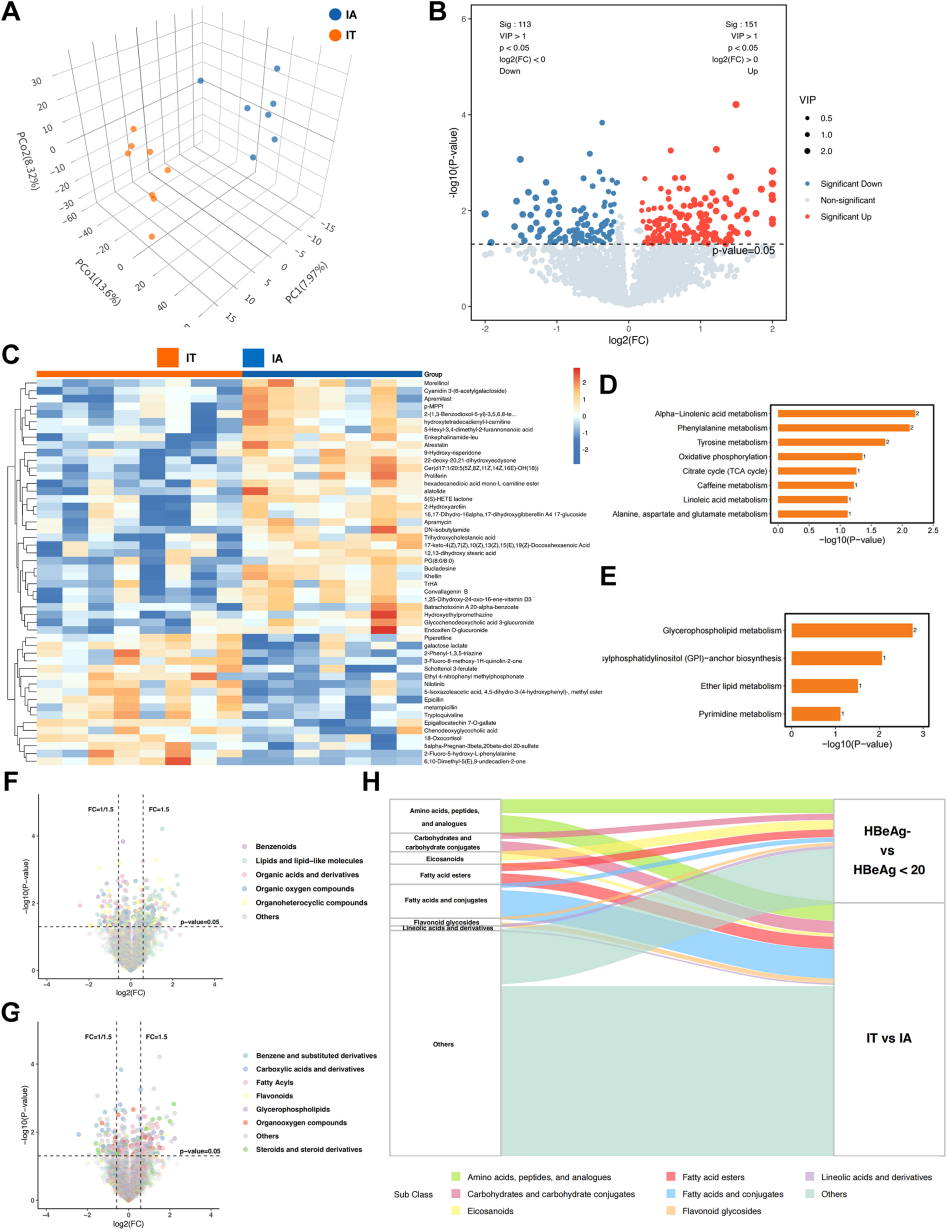
Altered metabolic pathways in immune tolerant and immune active patients. **(A)** OPLS-DA model of plasma metabolomic analysis for immune tolerant and immune active patients. **(B)** Volcano plot showing significantly upregulated and downregulated metabolites in immune tolerant and immune active patients. **(C)** Heatmap of differential metabolites between the immune tolerant and immune active groups. KEGG pathway enrichment analysis identified downregulated pathways **(D)** and upregulated pathways **(E)** in immune tolerant patients compared with immune active patients. **(F)** Volcano plot showing significantly regulated metabolites at the superclass level and **(G)** at the class level. **(H)** Alluvial diagram demonstrating divergent differential metabolites in patients with different HBeAg levels and immune phases.

Given that the IT and IA are crucial immune phases in HBeAg-positive CHB patients, we examined metabolic profiles in the IT and IA groups. The IT group displayed distinct metabolic profiles compared with the IA group (**Figure 4A**). A total of 264 dysregulated metabolites were identified in IT patients, including 113 downregulated and 151 upregulated metabolites (**Figures 4B, C**). KEGG pathway enrichment analysis revealed 4 dysregulated metabolic pathways. In particular, alpha-linolenic acid, phenylalanine, and tyrosine metabolism were downregulated, whereas glycerophospholipid metabolism was upregulated in IT patients (**Figures 4D, E**). Moreover, lipids and lipid-like molecules, especially steroids and fatty acyls, were significantly upregulated in IT patients compared with IA patients (**Figures 4F, G**). Immune clearance is a critical process for achieving HBeAg seroconversion. Thus, we sought to identify the differential metabolic characteristics that determine disease progression towards immune tolerance or HBeAg seroconversion. An alluvial diagram demonstrated that fatty acids and eicosanoids are the major differential metabolites that determine disease progression (**Figure 4H**).

### A predictive model integrating plasma gamma-glutamylglycine for HBeAg seroconversion

**Figure 5:**
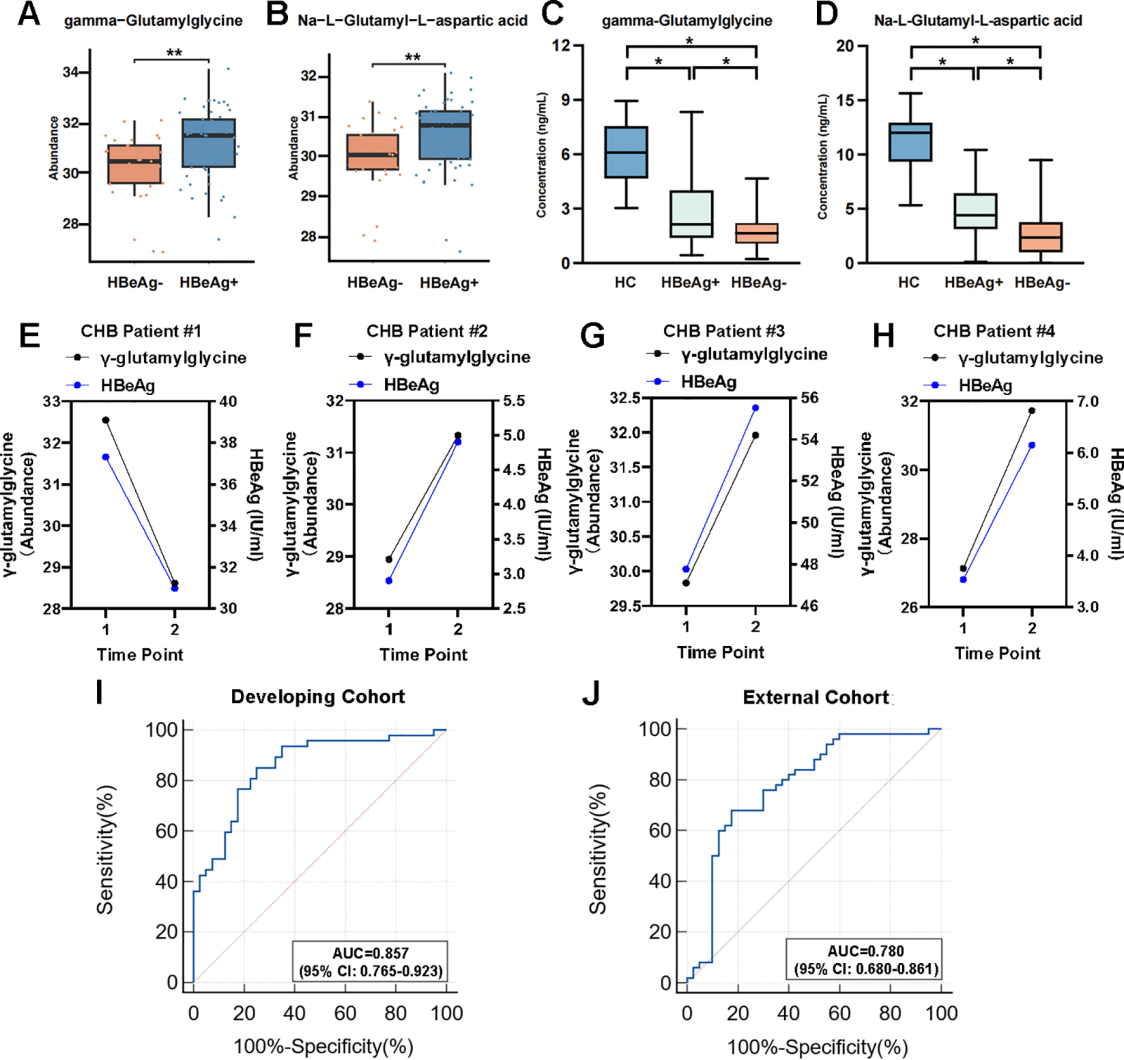
Performance of metabolite-based model for predicting HBeAg seroconversion. **(A, B)** Plasma levels of specific peptides were significantly altered in HBeAg-positive patients in developing cohort. **(C, D)** Plasma levels of specific amino acids and peptides were significantly altered in HBeAg-positive patients in external cohort. **(E-H)** Dynamic levels of gamma-glutamylglycine in some HBeAg-positive patients in developing cohort. **(I)** Receiver operating curve of metabolite-based model for predicting HBeAg seroconversion in developing cohort. **(J)** Receiver operating curve of metabolite-based model for predicting HBeAg seroconversion in external cohort. *P<0.05, **P<0.01.

Among the dysregulated amino acids and peptides, upregulated gamma-glutamylglycine and Na-L-glutamyl-aspartic acid were characterized in HBeAg-positive patients compared with those with HBeAg seroconversion (**Figures 5A, B**). The results were further verified by targeted metabolomic analysis in external cohort (**Figures 5C, D**). Moreover, dynamic analysis showed parallel change of plasma gamma-glutamylglycine and HBeAg in some HBeAg-positive CHB patients with negative serum HBV DNA who had been receiving NA treatment (**Figures 5E–H**). To develop a model for predicting HBeAg seroconversion, we constructed a random forest classifier based on differential peptides identified in the developing cohort. Accurate prediction was achieved using the model consisting of gamma-glutamylglycine and Na-L-glutamyl-aspartic acid, as indicated by an AUC of 0.857 (95% CI: 0.765-0.923) (**Figure 5I**). The sensitivity was 0.851 and the specificity was 0.750 in developing cohort. The model was further verified in an external cohort, in which plasma levels of gamma-glutamylglycine and Na-L-glutamyl-aspartic acid was measured by targeted metabolomic analysis. As shown in **Figure 5J**, the AUC of the predictive model was 0.780 (95% CI: 0.680-0.861) with a sensitivity of 0.680 and a specificity of 0.825 in external cohort. These results indicate that the predictive model based on plasma levels of gamma-glutamylglycine and Na-L-glutamyl-aspartic acid is sufficient to identify HBeAg seroconversion in HBeAg-positive patients, highlighting the potential of metabolomics for noninvasive prediction of HBeAg seroconversion.

### *In vitro* validation of the potential role of gamma-glutamylglycine

**Figure 6:**
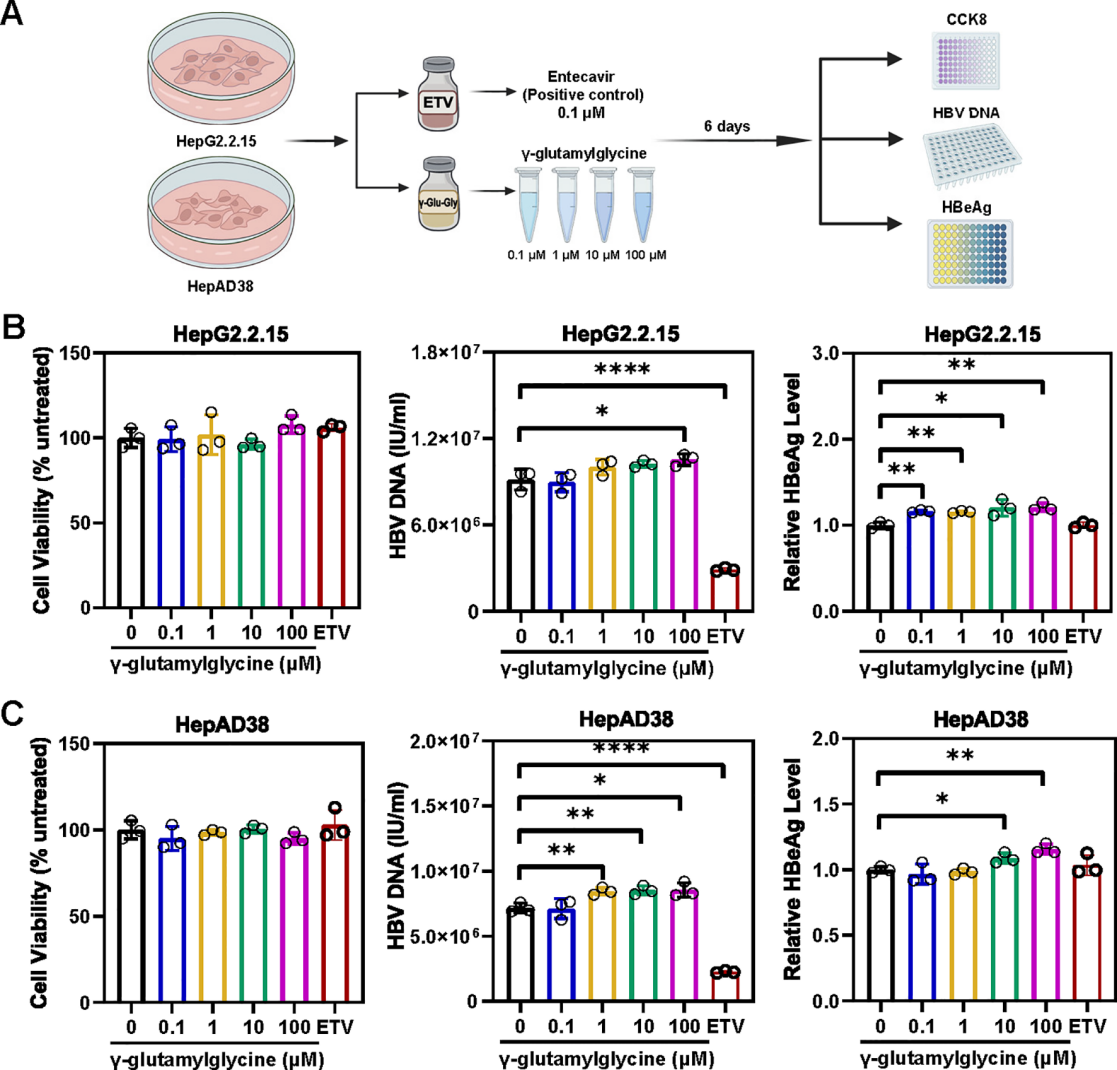
Role of gamma-glutamylglycine in HBV-replication cells. **(A)** Schematic workflow of the anti-HBV activity evaluation of gamma-glutamylglycine in HepG2.2.15 and HepAD38 cells. **(B)** HepG2.2.15 cells were treated with 0.1-100μM gamma-glutamylglycine for 6 days. CCK-8 assay was conducted, extracellular HBV DNA level and extracellular HBeAg level were measured. **(C)** HepAD38 cells were treated with 0.1-100μM gamma-glutamylglycine for 6 days. CCK-8 assay was conducted, extracellular HBV DNA level and extracellular HBeAg level were measured. *P<0.05, **P<0.01, ****P<0.0001.

We further investigated the potential functions and mechanisms of gamma-glutamylglycine using the HBV-replicating cell lines HepG2.2.15 and HepAD38 (**Figure 6A**). As shown in **Figures 6B, C**, the CCK-8 assay showed that gamma-glutamylglycine did not affect cell viability with a concentration range of 0.1-100μM in both HepG2.2.15 and HepAD38 cell lines. And gamma-glutamylglycine treatment was able to elevate the levels of HBV DNA and HBeAg in HepG2.2.15 and HepAD38 cells. These data suggested that gamma-glutamylglycine may play a role in the HBV replication cycle and contribute to the production of HBeAg, which is consistent with our metabolomic data.

## Discussion

Although antiviral drugs, such as PEG-IFNα and NAs, are available for treating HBeAg-positive CHB, achieving HBeAg seroconversion remains challenging for a large proportion of patients. In this study, we analyzed the metabolic characteristics of HBeAg-positive patients using untargeted and targeted metabolomic analysis and found that amino acid metabolism was prominently altered. These findings provide novel insights into the influence of metabolic status on HBeAg seroconversion and have important clinical implications for the management of HBeAg-positive patients.

One of the major findings of the present study is the identification of altered amino acid metabolism as a significant metabolic characteristic in HBeAg-positive patients. Several lines of evidence support the crucial role of amino acid metabolism in HBeAg seroconversion. First, KEGG pathway enrichment analysis revealed that amino acid metabolism was dysregulated not only in patients with HBeAg positivity but also in those with a low HBeAg level who failed to achieve HBeAg seroconversion. Second, multiple amino acid and peptide species were dysregulated in both the HBeAg-positive and HBeAg-low groups, with most amino acid and peptide levels being significantly lower in the HBeAg-positive group than in the HBeAg-negative group. Third, an metabolite-based model was sufficient to predict HBeAg seroconversion in HBeAg-positive patients. These results indicate that impaired amino acid metabolism may contribute to the failure of HBeAg seroconversion in HBeAg-positive patients. The alterations of amino acid metabolism have been implicated in different phases and progression of CHB. One study that included patients with various stages of hepatitis B infection demonstrated that the plasma levels of the amino acids threonine, glutamate, and methionine were increased while the plasma level of proline was decreased in patients with acute hepatitis B compared with healthy controls. In CHB patients, threonine, glutamate, methionine, and phenylalanine levels were increased, whereas valine, isoleucine, and leucine levels were decreased (18). Another metabolomic study demonstrated that amino acid metabolism was altered during progression from CHB to HCC, with L-serine and glycine distinguishing liver cirrhosis from CHB and L-serine distinguishing HCC from liver cirrhosis (19). Furthermore, it has been shown that the tryptophan ratio, branched-chain amino acids (BCAA)/aromatic amino acids ratio, BCAAs/tyrosine ratio, and serotonin-to-tryptophan ratio decreased while the tyrosine ratio and the kynurenine-to-tryptophan ratio increased in HCC patients compared to those in CHB (20). Consistent with these findings, our data indicate that alterations in amino acid and peptide metabolism represent a prominent metabolic characteristic in HBeAg-positive patients.

T-cell function is critical for antiviral immunity and HBV clearance in chronic HBV infection. Amino acid metabolism regulates T-cell function under various pathological conditions (21, 22). For instance, the essential amino acid methionine and the semi-essential amino acid cysteine, two sulphur-containing amino acids, are involved in regulating T cells. Because T cells lack the enzyme needed to convert methionine to cysteine, cysteine must be imported to support T-cell proliferation. Providing cysteine stimulates T-cell activity, whereas sequestering it has a suppressive effect (23). Similarly, serine directly affects T-cell proliferation by supplying one-carbon units for *de novo* nucleotide biosynthesis and generating S-adenosylmethionine, which supports histone methylation to regulate the production of cytokines, such as interleukin-1β (24). Glutamine is another amino acid whose depletion affects immune response balance. Glutamine favours a regulatory T-cell phenotype over a Th1 phenotype, rendering the immune response more suppressive, even in a cytokine environment that typically induces a Th1 phenotype (25). Therefore, glutamine supplementation has shown antiviral activity and holds potential as an adjuvant in modulating antiviral immune functions through interferon gamma signalling (26, 27). These findings indicate the potential for regulating T-cell function through targeting amino acid metabolism and suggests mechanisms underlying the regulatory role of amino acid metabolism in anti-HBV immunity and HBeAg seroconversion. Furthermore, amino acids and peptides may have a role in the HBV replication cycle. As indicated by our *in vitro* assays, gamma-glutamylglycine promote the levels of HBV DNA and HBeAg in HepG2 and HepAD38 cells. However, the effect of gamma-glutamylglycine on other HBV markers such as HBsAg and HBcAg should be further explored by *in vivo* and *in vitro* experiments to better illustrate the role of gamma-glutamylglycine in HBV replication cycle. Moreover, whether the physiological concentrations of gamma-glutamylglycine could reach the effective threshold should be further validated *in vivo* as the plasma and liver microenvironment concentration ranges of gamma-glutamylglycine could be varied.

Several approaches are available for predicting HBeAg seroconversion in HBeAg-positive patients. HBV-related biomarkers can serve as predictors of HBeAg seroconversion. For example, a study involving HBeAg-positive patients in the IA phase with a follow-period of up to 76 weeks without antiviral treatment demonstrated that HBV pgRNA is a reliable predictor of spontaneous HBeAg seroconversion (28). Similarly, serum levels of HBV RNA, HBsAg, and HBcrAg have been identified as predictors of HBeAg clearance and seroconversion in CHB patients treated with NAs (29–35). However, these HBV-related markers are considerably affected by antiviral therapy and may be less effective in patients receiving long-term antiviral treatment. In this study, we constructed a metabolite-based model to predict HBeAg seroconversion by performing untargeted metabolomic analysis to screen for differential metabolites in HBeAg-positive patients undergoing long-term antiviral therapy and further validated the model by targeted metabolomic analysis in an independent cohort. With the rapid development of multi-omics approaches, novel strategies for predicting HBeAg seroconversion are being developed (36–39). Integration of multiple biomarkers may allow for more accurate prediction of HBeAg seroconversion and enable personalized management strategies for HBeAg-positive patients.

This study has several strengths. First, we performed both untargeted metabolomic analysis to screen for differential metabolites associated with HBeAg seroconversion, offering a comprehensive view of metabolic profiles and targeted metabolomic analysis to validate the alterations of key amino acids and peptides. Second, we conducted a subgroup analysis focusing on patients with a low HBeAg level who had failed to achieve HBeAg seroconversion despite long-term NA therapy. Our findings indicate that altered amino acid metabolism is a key metabolic characteristic in these patients, potentially serving as an effective target for clinical intervention. Third, we explored the function of identified peptide and discovered that gamma-glutamylglycine increased HBV DNA and HBeAg levels in HBV-replicating cells. Moreover, we constructed a predictive model based on differential peptides to forecast HBeAg seroconversion and further validated its predictive performance in an independent cohort, providing a noninvasive approach to inform clinical management.

This study also has several limitations. First, although we included an external cohort to testify the predictive performance of the model constructed by developing cohort, the results should be further validated across different ethnicities and antiviral treatment. Second, the sample size of our subgroup analyses was limited and the results should be further validated in prospective cohorts with larger sample size. Third, although we have conducted *in vitro* assays to demonstrate the potential function of gamma-glutamylglycine in HBV replication, the investigations were preliminary and the exact mechanisms require further exploration.

## Conclusions

We analyzed the metabolic characteristics of HBeAg-positive patients and identified altered amino acid metabolism as a prominent feature, particularly in patients with low HBeAg levels. Gamma-glutamylglycine was identified as a metabolite that may hinder HBeAg seroconversion, and the metabolite-based model accurately predicted HBeAg seroconversion. These findings highlight the role of amino acid metabolism in HBeAg seroconversion and provide novel insights for the management of HBeAg-positive patients.

## Data Availability

The original contributions presented in the study are included in the article/supplementary material. Further inquiries can be directed to the corresponding author.
